# The Differences Between Multifocal and Unifocal Papillary Thyroid Carcinoma in Unilateral Lobe: A Meta-Analysis

**DOI:** 10.3389/fonc.2021.657237

**Published:** 2021-09-16

**Authors:** Ting Zhang, Liang He, Zhihong Wang, Wenwu Dong, Wei Sun, Ping Zhang, Hao Zhang

**Affiliations:** Department of Thyroid Surgery, The First Hospital of China Medical University, Shenyang, China

**Keywords:** unilateral unifocal PTC, unilateral multifocal PTC, central lymph node metastasis, lateral lymph node metastasis, tumor-node-metastasis stage, recurrence/persistence

## Abstract

**Background:**

As many inconsistent reports on the clinical manifestations and prognosis between unilateral unifocal PTC (UUPTC) and unilateral multifocal PTC (UMPTC), identifying the difference should guide management. The purpose of this study was to investigate other additional differences between UUPTC and UMPTC in addition to their difference in the number of cancer foci.

**Data Sources:**

A systematic literature search was conducted in the PubMed and Web of Science databases for relevant studies published before December 31, 2020. Their reference lists were also reviewed.

**Review Methods:**

Two reviewers independently extracted data and assessed the quality of eligible studies. Studies on patients who underwent an open thyroidectomy with or without neck dissection were included. Data were analyzed using the RevMan 5.3 software.

**Results:**

Fifteen studies comprising 9,665 patients were selected for the meta-analysis. UMPTC occurred in 10% to 36% of all PTC cases. There were no significant differences between UMPTC and UUPTC patients in age, gender, tumor size, and extrathyroidal extension (ETE). However, significant differences (P < 0.05) between UMPTC and UUPTC patients were observed in central lymph node metastasis (CLNM), lateral lymph node metastasis (LLNM), tumor-node-metastasis (TNM) stage I+II, TNM stage III+IV, the recurrence/persistence of the UMPTC group after total thyroidectomy and overall recurrence/persistence.

**Conclusion:**

UMPTC patients are more likely to have CLNM, LLNM, more advanced TNM stage, and recurrence/persistence than UUPTC patients. Compared with UUPTC, UMPTC patients should undergo central lymph node dissection, and pay more attention to LLNM, TNM stage and recurrence/persistence during the follow-up.

## Introduction

Papillary thyroid carcinoma (PTC) is the most common endocrine malignancy (1). It is categorized into unilateral PTC and bilateral PTC based on tumor location and into unifocal PTC and multifocal PTC based on the number of cancer foci. Multifocality is a common pathological feature of PTC. However, the prevalence of multifocality is significantly lower than that of unifocality in the unilateral lobe. To date, the etiology of multifocal thyroid cancer remains unclear.

There have been many different basic research reports on the occurrence of multifocal PTC in recent years, some believed to be of polyclonal origin (2, 3), but others considered to be intraglandular spread (4, 5). It is postulated that the clinical manifestations of bilateral PTC are worse than unilateral PTC. The bilateral PTC has multiple cancer foci, easy extrathyroidal extension (ETE) (6), uncomplicated cervical lymph node metastasis, easy recurrence, and poor prognosis after the operation (7). Cognizant of this, radical resection treatment is needed, followed by a close follow-up after surgery. However, there are different treatment opinions for unilateral PTC. Unilateral unifocal PTC (UUPTC) can be surgically ablated (8) though some scholars choose to carry out active surveillance (9). Both the American Thyroid Association (ATA) and the National Comprehensive Cancer Network (NCCN) recommend total surgical thyroidectomy for unilateral multifocal PTC (UMPTC) (10, 11).

Nonetheless, there are many inconsistent reports on the clinical manifestations and prognosis between UUPTC and UMPTC.

Some studies postulate that the prognosis of UMPTC is poor (12), whereas others suggest that UMPTC has good prognosis as UUPTC because they are differentiated thyroid carcinoma (13, 14). Herein, a meta-analysis was performed to investigate the differences between multifocal and unifocal PTC in the unilateral lobe.

## Materials and Methods

This meta-analysis was carried out following the Preferred Reporting Items for Systematic Reviews and Meta-analysis (PRISMA) guidelines (15).

### Search Strategy

A systematic literature search of relevant studies published before December 31, 2020, was performed in the PubMed and Web of Science databases. The key words included (((thyroid cancer OR thyroid carcinoma) AND papillary) OR PTC) AND [(Multifocal OR Multiple OR Multinodular) AND (Unifocal OR Solitary OR Single) AND (Unilateral OR one side lobe)]. Two authors (Zhang T and Sun W) conducted the selection process independently. All discrepancies were resolved through discussions and consensus by the two authors or referred to a third author.

### Selection Criteria

Prospective or retrospective studies published in English and whose participants were primary PTC patients who underwent thyroid and lymphadenectomy surgery were included in the meta-analysis. The included studies had their participants diagnosed through intraoperative or postoperative pathology. Moreover, the studies had extractable demographics and clinical data for thyroidectomy patients. However, review articles, conference abstracts, editorials, letters, and single case reports were excluded. Duplicate studies and those with no reported outcomes were also excluded.

### Data Extraction and Quality Assessment

**Figure 1:**
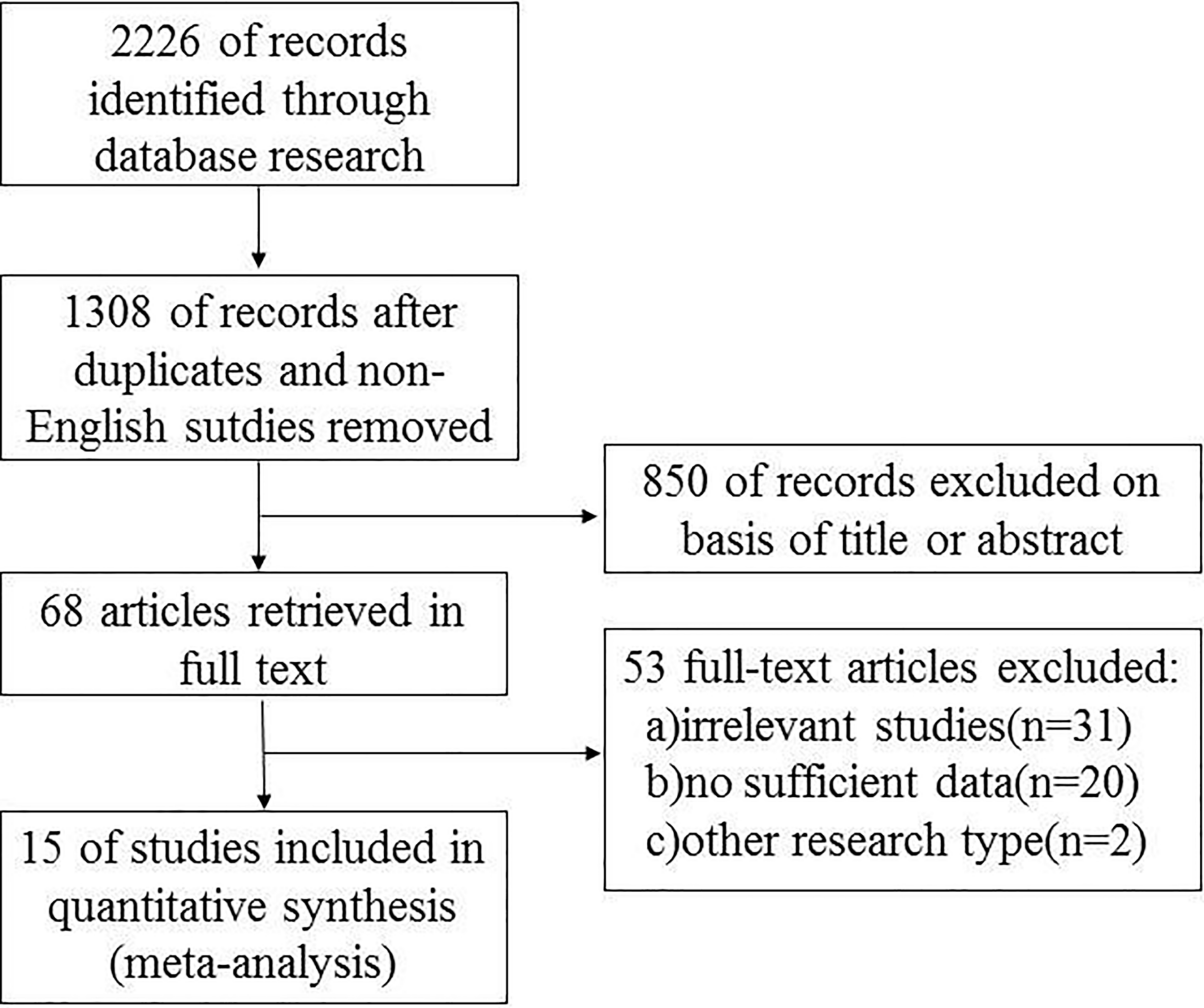
Flowchart of study selection.

Relevant data from the included articles were extracted independently by the two investigators following a standardized format. The data included the first author’s name, year of publication, country of origin, research design, number of cases, potential risk factors, and other corresponding data (**Figure 1**). The potential risk factors included age, gender, extrathyroidal extension (ETE), central lymph node metastasis (CLNM), lateral lymph node metastasis (LLNM), and the tumor-node-metastasis (TNM) stage and recurrence/persistence. The Newcastle-Ottawa quality assessment scale was used to assess the quality of the studies (16).

### Data Analysis

Data analysis was performed using Review Manager version 5.3 (Cochrane Collaborative, Oxford, United Kingdom), and results presented as the mean difference IV (MD) or odds ratios (ORs) with a 95% confidence interval (CI). A P-value of less than 0.05 indicated that there was a statistical significance of the observed difference. Heterogeneity of the data was quantified using the Q test and I^2^ statistics, while that among studies was estimated using Cochran’s Q statistic (17). The fixed-effects model was used when P > 0.10 and I^2^ < 50%; otherwise, a random-effects model was applied. The potential publication bias was evaluated using Begg’s funnel plot.

## Results

The search strategy generated 2226 potentially relevant studies for meta-analysis. **Figure 1** is a flowchart detailing the studies retrieved and excluded. After removing studies that did not meet the inclusion criteria,15 studies (7, 14, 18–30) comprised of 9665 patients were selected for analysis. Amongst the studies, the incidence of multifocal and unifocal PTC was 10% - 36% and 63% - 90%, respectively. The incidences of all the studies are summarized in **Table 1**.

**Table 1:** Characteristics of eligible studies.

Author	Year	Country	Study design	Case number		Quality score
				Multifocal	Unifocal	
Kalliopi Pazaitou-Panayiotou	2008	Greece	retrospective analysis	10	44	6
Y. C. Lim	2009	korea	retrospective analysis	9	77	7
Hye Jeong Kim	2013	korea	retrospective analysis	193	1423	9
Qunzi Zhao	2013	china	retrospective analysis	23	140	8
XIAOLONG LI	2013	china	retrospective analysis	35	312	8
Ayham Al Afif MSc	2015	Canada	retrospective analysis	46	85	8
Kuk-Jin Kim	2015	korea	retrospective analysis	821	1488	9
Abbas Ali Tam	2016	Turkey	retrospective analysis	121	604	8
Ning Qu	2016	china	retrospective analysis	78	287	8
Weibin Wang	2016	china	retrospective analysis	211	1517	9
Hai-Jiang Qu	2017	china	retrospective analysis	111	302	8
Tinghai Xiang	2018	china	retrospective analysis	40	147	7
Krzysztof Kaliszewski	2019	Poland	retrospective analysis	48	114	7
Yossi Geron	2019	Israel	retrospective analysis	156	505	8
Victoria Harries	2020	USA	retrospective analysis	230	619	9

### Age

**Figure 2:**
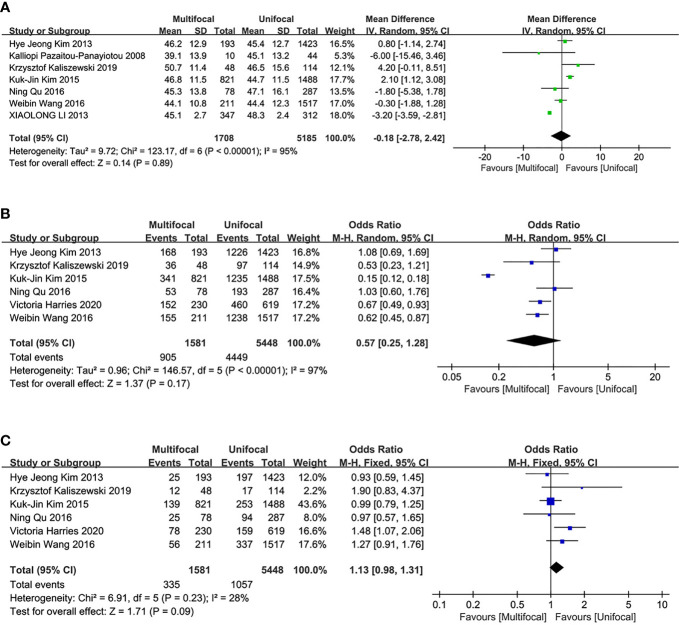
Meta-analysis results for the difference between the UMPTC and UUPTC. **(A)** Age; **(B)** Female; **(C)** Male.

Seven studies were included in the analysis of differences between UMPTC and UUPTC patients based on age. There were no significant differences between the UMPTC and UUPTC patients based on age (MD = -.018, 95% CI = -2.78–2.42, P = 0.89) (**Figure 2A**).

### Gender

Six studies were included in the analysis of the differences between UMPTC and UUPTC patients based on gender (male and female). There were no significant differences in the number of male and female patients between the two groups (male, OR = 1.13, 95% CI = 0.98–1.31, p = 0.09; female, OR = 0.57, 95% CI = 0.25–1.28, p = 0.17) (**Figures 2B, C**).

### Tumor Size

**Figure 3:**
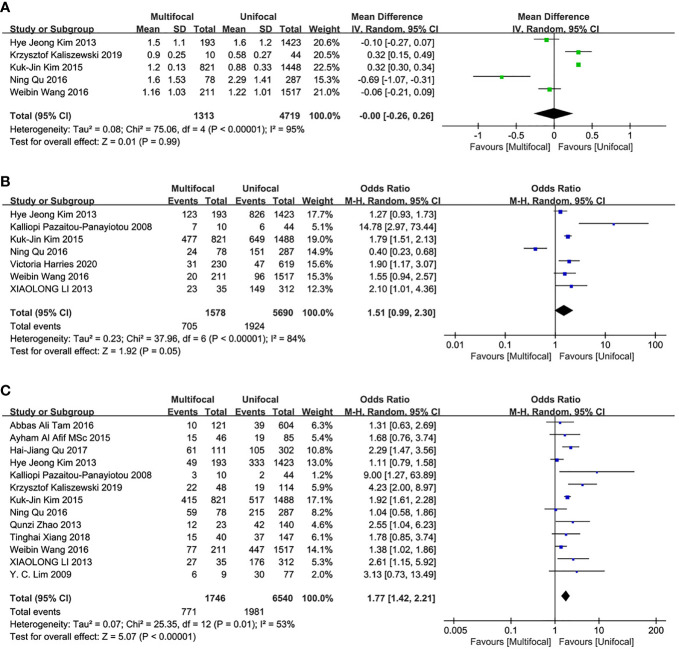
Meta-analysis results for the difference between the UMPTC and UUPTC. **(A)** Size; **(B)** ETE; **(C)** CLNM.

Five studies were included in this analysis. There were no significant differences in the primary tumor size between UMPTC and UUPTC patients (OR = 0, 95% CI = -0.26–0.26, p = 0.99) (**Figure 3A**).

### Incidences of ETE

This analysis included seven studies. There were no significant differences in ETE incidences between UMPTC and UUPTC patients (OR = 1.51, 95% CI = 0.99–2.30, P = 0.05) (**Figure 3B**).

### Incidences of CLNM

Thirteen studies were included in the analysis of differences between UMPTC and UUPTC patients based on CLNM. There were significant differences in CLNM incidences between the two groups. The UMPTC group had higher incidences of CLNM than the UUPTC group (OR = 1.77, 95% CI = 1.42–2.21, P < 0.00001) (**Figure 3C**).

### Incidences of LLNM

**Figure 4:**
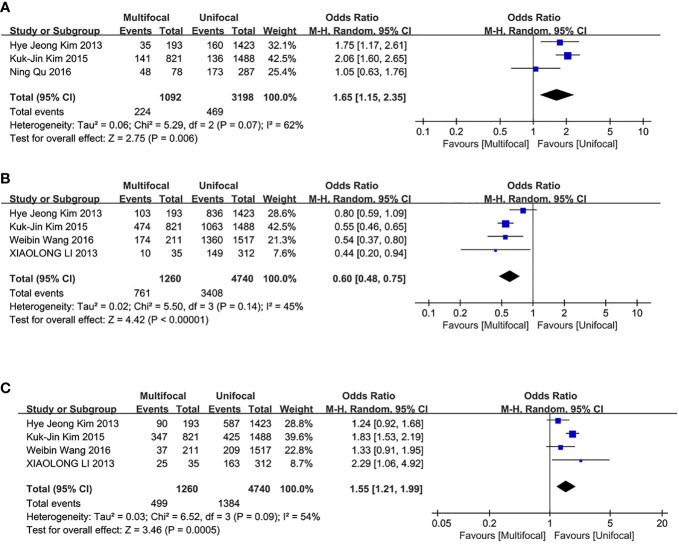
Meta-analysis results for the difference between the UMPTC and UUPTC. **(A)** LLNM; **(B)** TNM stage I+II; **(C)** TNM stage III+IV.

This analysis included three studies. LLNM incidences were significantly different between the two groups. The UMPTC group had higher incidences of LLNM than the UUPTC group (OR = 1.65, 95% CI = 1.15–2.35, p = 0.006) (**Figure 4A**).

### TNM Stage

The analysis included four studies. The UMPTC group exhibited significantly less TNM stage I+II than the UUPTC group (OR = 0.60, 95% CI = 0.48–0.75, P < 0.00001) (**Figure 4B**). However, it exhibited significantly higher TNM stage III+IV than the UUPTC group (OR = 1.55, 95% CI = 1.21–1.99, p = 0.0005) (**Figure 4C**).

### Recurrence/Persistence of PTC

**Figure 5:**
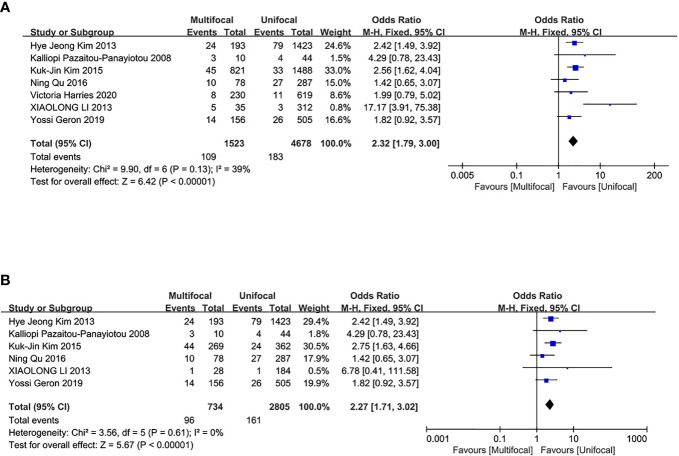
Meta-analysis results for the difference between the UMPTC and UUPTC. **(A)** Overall recurrence/persistence; **(B)** The recurrence/persistence after total thyroidectomy.

The overall recurrence/persistence of PTC was assessed in seven studies. The UMPTC group had a higher incidence of overall PTC recurrence/persistence compared to the UUPTC group (OR = 2.32, 95% CI = 1.79–3.00, P < 0.00001) (**Figure 5A**). Analysis of PTC recurrence/persistence between the two groups after total thyroidectomy revealed that the UMPTC group had a significantly higher recurrence/persistence than the UUPTC group (OR = 2.27, 95% CI = 1.71–3.02, P < 0.00001) (**Figure 5B**). Six studies were included in this analysis.

## Discussion

Studies hypothesize that multifocality in PTC results from intrathyroidal metastasis from the primary tumor or the coexistence of separate neoplastic foci (31, 32). Testing the latter hypothesis revealed that BRAFV600E mutation is a risk factor for PTC multifocality (33). In the same line, the clinical and prognostic implications of multifocality *vs.* unifocality for PTC patients are still controversial. This study is the first meta-analysis to explore the difference between UMPTC and UUPTC patients.

Several studies postulate that some clinicopathological features are different between UMPTC and UUPTC, including age, gender, tumor size, and ETE. Qu N et al. (7) reported that an increase in tumor foci during diagnosis was strongly associated with older age. Similarly, Emine Ozlem Gur found out that the tumor size of the multifocal group was significantly larger than that of the unifocal group in a study that comprised 305 PTC patients (34). It is also postulated that females have higher incidences of UMPTC compared to males (35). A retrospective analysis involving 2,095 PTC patients reported that multifocal tumors (32% of cases) had a significantly higher ETE incidence than unifocal tumors (20). However, other studies report that the tumor characteristics of UMPTC and UUPTC are similar (36, 37).

The association between CLNM and LLNM with UMPTC and UUPTC remains controversial. Some studies postulate that multifocality is not independently associated with CLNM (38). However, many studies have focused on the risk factors of CLNM and LLNM for UMPTC patients. For example, Zheng WH suggests that multifocality should be assessed when selecting patients for prophylactic central neck lymph node dissection (39). Multivariate analysis by Luca Sessa and Yoon Kyoung So revealed that multifocality is an independent risk factor for CLNM and LLNM (40, 41). Besides, tumor classification was standardized using the TNM stage system of the American Joint Committee on Cancer. The TNM stage system is used to stage thyroid cancers. It can then be used in combination with patient characteristics to define likely prognosis (42). Although most TNM stage systems do not include PTC multifocality, many endocrine surgeons regard it as an important factor in patient treatment. Kim et al. reported that an increase in tumor foci was strongly associated with the advanced TNM stage of PTC. The advanced TNM stage is significantly more frequent in UMPTC than in UUPTC (43). Li Genpeng et al. reported that patients with multifocal PTC had a more advanced TNM stage (44).

In the 2015 update of the ATA guidelines, multifocality was not regarded as a risk factor in the three-tiered categorical risk of recurrence system. Some studies report that PTC patients have recurrent cases despite the general thought that PTC has an excellent prognosis. Previous studies have contradicting opinions about whether the recurrence/persistence of PTC is different between UMPTC and UUPTC. Recently, a multicentered study comprised of a large cohort demonstrated that tumor multifocality lacks an independent risk prognostic value in clinical outcomes of UMPTC (45). However, some studies report that UMPTC patients have a higher risk of recurrence/persistence than UUPTC patients (46, 47).

In our study, there were no significant differences in age, gender, size, and ETE between the UMPTC and UUPTC patients, which was inconsistent with these published results, these results may have been caused by differences in the selection criteria and different study designs. However, our analysis showed UMPTC patients were more likely to have CLNM, LLNM and advanced TNM stage during diagnosis compared to UUPTC patients. The possible reason is that UMPTC is associated with tumor aggressiveness, and UMPTC plays an important role in tumorigenesis and development.

Clinical recurrences/persistence included local recurrence/persistence, contralateral residual lobe, the cervical lymph nodes, and distant metastasis. The operative methods of overall recurrence/persistence rate included total thyroidectomy, near-total thyroidectomy, hemithyroidectomy, and subtotal thyroidectomy. The recurrence/persistence of total thyroidectomy between UMPTC and UUPTC was further analyzed to eliminate the interference of other operative methods on the overall recurrence/persistence of the contralateral remnant lobe. Differences in recurrence/persistence of total thyroidectomy were found to be similar to the overall recurrence. The recurrence/persistence of UMPTC after total thyroidectomy was higher than UUPTC. A forest map analysis of recurrence/persistence after other operative methods was not made because of inadequate information. Nonetheless, regardless of the surgical extent, UMPTC is more likely to have recurrence/persistence than UUPTC. Consequently, clinicians need to employ more aggressive initial treatment and closer follow-up for patients with UMPTC. Herein, it was concluded that UMPTC patients have a higher incidence rate of overall recurrence/persistence than UUPTC patients.

Despite the significant positive findings, this study was limited by several factors. The number of studies included was small because of the unavailability of raw data from some articles. Moreover, most of the patients in the included studies were from Asia, which may cause bias if the findings are applied in all races. There was no randomized controlled trial included in this study. In the same line, this meta-analysis included several large studies that could have introduced some bias in general study outcomes. The types of patients that underwent thyroidectomy for thyroid lesions were inconsistent, including total thyroidectomy, subtotal thyroidectomy, and lobectomy plus isthmus. Besides, differences in the study populations and aims of the included studies might have led to selective bias. Only the number of patients with or without recurrence/persistence were counted without distinguishing the site of recurrence/persistence, ipsilateral or bilateral, synchronous or metachronous, and single or multiple time/site recurrence. Lateral cervical nodes were dissected only in patients suspected of having lymph node metastasis based on clinical or radiologic examinations in most of the studies. Patients who had not undergone LLN dissection were regarded as LLN negative. Therefore, the rate of LLNM might have been underestimated.

## Conclusions

The prevalence of UMPTC is significantly lower than UUPTC despite some clinicopathological features such as age, gender, tumor size, and ETE lacking significant differences between UMPTC and UUPTC patients. However, UMPTC patients are more likely to have CLNM, LLNM, more advanced TNM stage, and recurrence/persistence than UUPTC patients. Therefore, compared with UUPTC, UMPTC patients should undergo central lymph node dissection, and pay more attention to LLNM, TNM stage and recurrence/persistence during the follow-up.

## Data Availability Statement

The raw data supporting the conclusions of this article will be made available by the authors, without undue reservation.

## Author Contributions

HZ provided the conception of the study and edited the final manuscript. TZ contributed significantly to manuscript design and preparation. TZ and WS finished clinical data collection and literature research. LH, ZW, WD, and PZ analyzed the data. All authors contributed to the article and approved the submitted version.

## Funding

This work was supported by the Scientific Research Foundation of The Education Department of Liaoning Province, China (grant no. QNZR2020009).

## Conflict of Interest

The authors declare that the research was conducted in the absence of any commercial or financial relationships that could be construed as a potential conflict of interest.

## Publisher’s Note

All claims expressed in this article are solely those of the authors and do not necessarily represent those of their affiliated organizations, or those of the publisher, the editors and the reviewers. Any product that may be evaluated in this article, or claim that may be made by its manufacturer, is not guaranteed or endorsed by the publisher.
